# Effects of butanol fraction of *Ziziphus mucronata* root ethanol extract on glucose homeostasis, serum insulin and other diabetes-related parameters in a murine model for type 2 diabetes

**DOI:** 10.1080/13880209.2016.1242632

**Published:** 2016-12-09

**Authors:** Mohammed Auwal Ibrahim, Md. Shahidul Islam

**Affiliations:** a School of Life Sciences, University of KwaZulu-Natal (Westville Campus), Durban, South Africa;; b Department of Biochemistry, Ahmadu Bello University, Zaria, Nigeria

**Keywords:** Buffalo thorn, β-cell function, liver glycogen, insulin resistance, blood glucose, glucose tolerance

## Abstract

**Context:***Ziziphus mucronata* Willd (Rhamnaceae) is currently used in Nigerian traditional treatment of diabetes mellitus. However, detailed information on the antidiabetic potential of the plant parts is presently unknown.

**Objectives:** The present study investigated the antidiabetic effects of the butanol fraction of *Z. mucronata* root (ZMBF) in a type 2 diabetes (T2D) model of rats.

**Materials and methods:** T2D was induced in rats by feeding a 10% fructose solution *ad libitum* for two weeks followed by an intraperitoneal injection of streptozotocin (40 mg/kg bw) and the animals were orally treated with ZMBF 150 or 300 mg/kg bw for five days a week for four weeks. Food and fluid intake, body weight changes and blood glucose levels were monitored during the experiment while other blood and organ specific diabetes-associated parameters were measured at the end of the experiment.

**Results:** After four-week treatment, significantly (*p* < 0.05) lower blood glucose (19.24 vs 28.96 mmol/L), improved glucose tolerance ability (21.26 vs 28.56 mmol/L), higher serum insulin (131.37 vs 64.20 pmol/L) and liver glycogen (2.40 vs 1.54 mg/g tissue) were observed in the 300 mg/kg ZMBF ingested group compared with the diabetic control group. However, food and fluid intake, body weight gain, HOMA-β, HOMA-IR, serum fructosamine level, hepatic and renal function tests were not significantly (*p* > 0.05) affected by the treatment of ZMBF.

**Conclusion:** Results of this study suggest that ZMBF treatment, at 300 mg/kg bw, possess antidiabetic activity, but could not ameliorate some diabetes-related parameters in type 2 diabetic rats.

## Introduction

The International Diabetes Federation (IDF) estimates that currently 415 million adults worldwide are affected by diabetes and this figure is expected to reach a staggering 642 million by 2040 (IDF, 2015) while type 2 diabetes (T2D) accounts for >90% of all the diabetic cases. T2D is a complex, heterogeneous and polygenic disease characterized mainly by insulin resistance and pancreatic β-cell dysfunction (Salas-Salvado et al. 2011), which leads to chronic hyperglycemia. Several mechanisms have been proposed for the β-cell destruction including oxidative stress (Wright et al. 2006). Currently, T2D is predominantly managed with sulfonylureas, biguanides and α-glucosidase inhibitors (Ghadyale et al. 2012). Unfortunately, the use of these synthetic antidiabetic agents is beset with a number of side effects such as diarrhoea, nausea and liver failure (Fujisawa et al. 2005).

In the last few years, there has been a growing interest in traditional herbal medicine for the care and management of diabetes both in developing and developed countries, due to their natural origin and propensity to elicit fewer side effects (Modak et al. 2007; Hasani-Ranjbar et al. 2008). Indeed, traditional medicinal plants were listed by the World Health Organization (WHO) expert committee on diabetes as one of the methods for the treatment of diabetes, which should be further investigated (Tabatabaei-Malazy et al. 2012) to provide potential chemical leads for the development of novel antidiabetic agents. In this context, African medicinal plants may provide the needed chemical leads because of their multiple health benefits.

*Ziziphus mucronata* Willd (Rhamnaceae), commonly referred to as ‘buffalo thorn’, is available in most parts of Africa. A decoction of the plant is used traditionally in the treatment of diabetes mellitus (Etuk et al. 2010) among the rural inhabitants of northern Nigeria. In spite of this report, the antidiabetic effects of the plant have not been scientifically validated either in humans or experimental animals. In fact, apart from a few reports (Mpiana et al. 2008; Waterman et al. 2010; Adewusi & Steenkamp 2011), information on the pharmacological activities of the plant is scarce in the literature. In a recent study (Ibrahim et al. 2012), we subjected the various crude solvents extracts from the stem bark, root and leaf samples of the plant to a series of *in vitro* antioxidative assays and reported that the ethanol extract of the root had the most potent antioxidative activities in multiple models of antioxidative assay.

Hence, in the present study, we conducted a comprehensive investigation on the *in vivo* activity of the butanol fraction of the *Z. mucronata* on T2D, an oxidative stress-related metabolic disorder.

## Materials and methods

### Plant material

The root samples of *Z. mucronata* were freshly collected in the month of January 2011 from Zaria, Kaduna state, Nigeria. The plant was identified and authenticated at the herbarium unit of the Biological Science Department, Ahmadu Bello University, Zaria, Nigeria, by Mr. Umar Gallah and a voucher specimen number 154 was deposited accordingly. The root samples were immediately washed and shade-dried for two weeks to constant weights. The dried samples were grounded to a fine powder, and then stored individually in airtight containers for transport to the University of KwaZulu-Natal, Westville campus, South Africa, for subsequent analysis.

### Extraction and solvent-partitioned fractionation

The fine powdered root (4 kg) was defatted with hexane then extracted with 10 L of ethanol by soaking for 48 h and filtered through Whatmann filter paper (No. 1). The resultant extract was evaporated in vacuum using a rotary evaporator (Buchi Rotavapor II, Buchi, Germany) at 40 °C to obtain the crude ethanol extract with a yield of 1.16%. The crude ethanol root extract (50 g) was dissolved in 600 mL of distilled water: methanol (9:1) and successively partitioned with hexane (2 × 500 mL), dichloromethane (2 × 500 mL), ethyl acetate (2 × 500 mL) and butanol (2 × 500 mL). The fractions were evaporated to dryness in vacuum at 40 °C under reduced pressure whereas the remaining aqueous fraction was dried in water bath at 50 °C. The fractions were transferred to micro tubes and stored at 4 °C until further analysis. The butanol fraction (ZMBF) displayed the highest α-glucosidase and α-amylase inhibitory effect (data not shown) amongst the fractions and therefore was subjected to further *in vivo* studies.

### Experimental animals

Six-week-old male Sprague–Dawley (SD) rats were obtained from the Biomedical Resource Unit (BRU) located at the University of KwaZulu-Natal (Westville Campus), South Africa with a mean initial body weight (bw) of 207.60 ± 4.27 g. Two animals were housed in one medium sized poly-carbonate cage in a temperature and humidity controlled room with a 12 h light-dark cycle. A standard rat pellet diet was supplied *ad libitum* during the entire experimental period and the animals were maintained according to the rules and regulations of the Experimental Animal Ethics Committee of the University of KwaZulu-Natal, South Africa (Ethical approval number: 022/12/Animal).

### Animal grouping and induction of type 2 diabetes

Animals were randomly divided into six groups of eight animals each namely: Normal Control (NC), Diabetic Control (DBC), Diabetic + low dose (150 mg/kg bw) of ZMBF (DZL), Diabetic + high dose (300 mg/kg bw) of ZMBF (DZH), Diabetic + metformin (300 mg/kg bw) (DMF), Non-diabetic + high dose (300 mg/kg bw) of ZMBF (NZT). After one week adaptation period and a subsequent overnight fast, the animals in DBC, DZL, DZH and DMF groups were supplied with a 10% fructose solution *ad libitum* for two weeks to induce insulin resistance followed by a single injection (i.p.) of streptozotocin (STZ) (40 mg/kg bw) dissolved in citrate buffer (pH 4.5) to induce partial pancreatic β-cell dysfunction. The animals in NC and NZT groups were supplied with normal drinking water and injected with citrate buffer instead of 10% fructose and STZ injections, respectively (Wilson & Islam 2012). One week after STZ injection, the non-fasting blood glucose (NFBG) levels of all animals were measured in the blood collected from the tail vein by using a portable glucometer (Glucoplus Inc., Saint-Laurent, Quebec, Canada). Animals with a NFBG level >18 mM were considered to be diabetic (Islam 2011a) while the animals with a NFBG level <18 mM were excluded from the study.

### Intervention trial

After the confirmation of diabetes (one week after the streptozotocin injection), a respective dose of the fraction was orally administered five days a week to the animals in the DZL and DZH and NZT groups by using gastric gavage needle. Animals in controls (NC and DBC) and DMF groups were treated with a similar volume of the vehicle and metformin (300 mg/kg bw), respectively, for a four-week intervention period. During this period, daily food and fluid intake as well as weekly body weight and NFBG were measured in all animal groups.

### Oral glucose tolerance test

To measure the glucose tolerance ability of each animal, the oral glucose tolerance test (OGTT) was performed in the last week of the four-week intervention period. In this test, a single dose of glucose solution (2 g/kg bw) was orally ingested into each animal and the subsequent levels of blood glucose were measured at 0 (just before glucose ingestion), 30, 60, 90 and 120 min after the dose of glucose.

### Collection of blood and organs

At the end of the experimental period, animals were euthanized by halothane anaesthesia and blood and organ samples were collected. The whole blood of each animal was collected via cardiac puncture and immediately preserved in a refrigerator until further processing. The blood samples were centrifuged at 3000 rpm for 15 min and serum from each blood sample was separated and preserved at −30 °C for further analysis. The liver was collected from each animal, washed with normal saline, wiped with filter paper, weighed and preserved at −30 °C until subsequent analysis. A small piece of pancreatic tissue from each animal was cut and placed in a 10% neutral buffered formalin solution and preserved at room temperature for histopathological study. The neutral buffered formalin of each pancreatic tissue sample was replaced weekly during the entire preservation period.

### Analytical methods

The serum insulin concentrations were measured by an enzyme-linked immunosorbent assay (ELISA) method using an ultrasensitive rat insulin ELISA kit (Mercodia, Uppsala, Sweden) in a multi plate ELISA reader (Biorad-680, BIORAD Ltd, Japan). The serum lipid profile, fructosamine, urea and creatinine concentrations as well as liver function enzymes; aspartate and alanine aminotransferases (AST and ALT) and alkaline phosphatase (ALP) were measured using an Automated Chemistry Analyzer (LabmaxPlenno, Labtest Co. Ltd, Lagoa Santa, Brazil) with commercial assay kits from the same company. Homeostatic model assessment (HOMA-IR and HOMA-β) scores are usually used to determine the level of insulin resistance (HOMA-IR) and the level of pancreatic β-cells function (HOMA-β). These were calculated using fasting serum insulin and FBG concentrations measured at the end of the intervention period according to the following formula:

$$\text{HOMA}-\text{IR}=\frac{\text{Fasting serum insulin in}\frac{\text{U}}{\text{L}}\times \text{ Fasting blood glucose in mmol}/\text{L }}{22.5}$$

$$\mathrm{HOMA}-\beta =\frac{20\;\times \;\mathrm{Fasting}\;\mathrm{serum}\;\mathrm{insulin}\;U/L}{\mathrm{Fasting}\;\mathrm{blood}\;\mathrm{glucose}\;\mathrm{in}\;\mathrm{mmol}/L-3.5\;}$$

Conversion factor: insulin (1U/L = 7.174 pmol/L)

Liver glycogen concentrations were measured by a phenol-sulphuric acid method as described by Lo et al. (1970).

### Histopathological examination of pancreatic tissue

The formalin preserved pancreatic tissues were treated according to a standard laboratory protocol for paraffin embedding. Sections were cut at a size of 4 μm. Then, slides were deparaffinized in *p*-xylene and rehydrated in changes of ethanol (100, 80, 70, 50%) and rinsed in water. Slides were stained in haematoxylin for 5 min and rinsed with water. Slides were counterstained in eosin, mounted in DPX (distyrene, tricresyl phosphate and xylene) cover-slipped and viewed with Leica slide scanner (SCN 4000, Leica Biosystems, Germany).

### Statistical analysis

All data were presented as the mean ± SD. Data were analyzed by using a statistical software package (SPSS for Windows, version 18, IBM Corporation, Armonk, NY) using Tukey’s-HSD multiple range *post hoc* test. Values were considered significantly different at *p <* 0.05.

## Results

**Figure 1.**
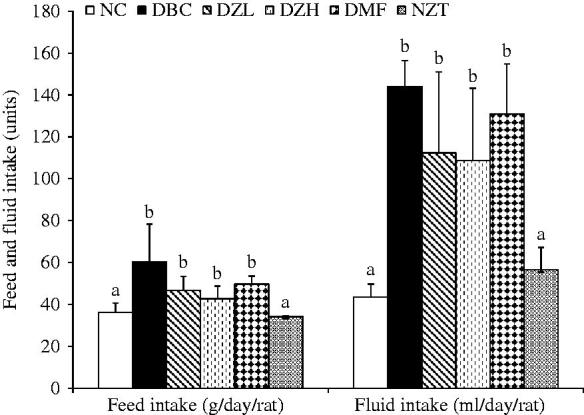
The effects of oral treatment of butanol fraction of *Z. mucronata* root on feed and fluid intakes of type 2 diabetic rats. Data are presented as the mean ± SD of eight animals. ^a–b^Values with different letters over the bars for a given parameter are significantly different from each other (Tukey’s-HSD multiple range *post hoc* test, *p* < 0.05). NC: Normal Control; DBC: Diabetic Control; DZL: Diabetic *Ziziphus mucronata* low dose (150 mg/kg bw); DZH: Diabetic *Ziziphus mucronata* high dose (300 mg/kg bw); DMF: Diabetic metformin; NZT: Normal *Ziziphus mucronata* toxicological dose (300 mg/kg bw).

**Figure 2.**
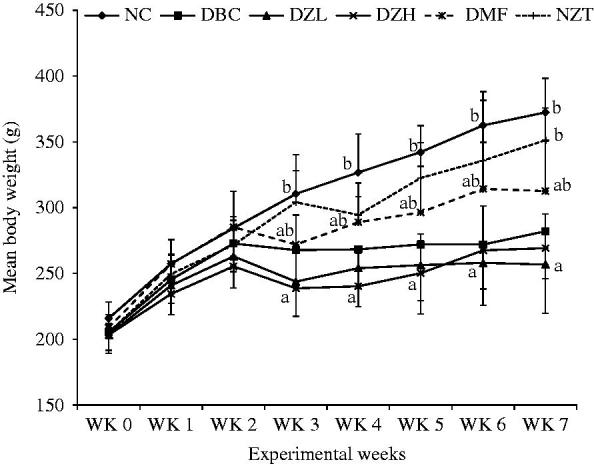
The effects of oral treatment of butanol fraction of *Z. mucronata* root on mean body weight gain of type 2 diabetic rats. Data are presented as the mean ± SD of eight animals. ^a–b^Values with different letters for a given week are significantly different from each other (Tukey’s-HSD multiple range *post hoc* test, *p* < 0.05). NC: Normal Control; DBC: Diabetic Control; DZL: Diabetic *Ziziphus mucronata* low dose (150 mg/kg bw); DZH: Diabetic *Ziziphus mucronata* high dose (300 mg/kg bw); DMF: Diabetic metformin; NZT: Normal *Ziziphus mucronata* toxicological dose (300 mg/kg bw).

**Figure 3.**
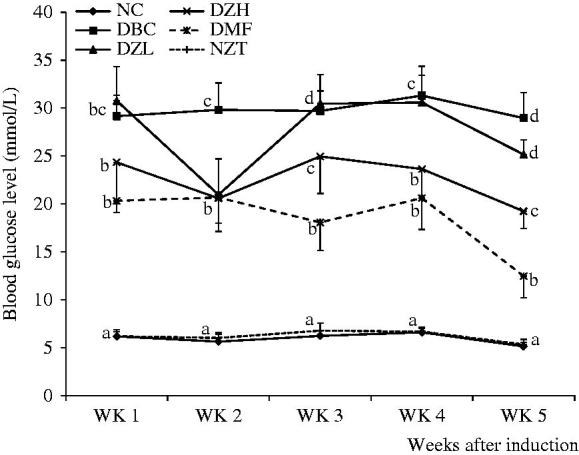
Weekly blood glucose concentrations (post induction) of different animal groups. Data are presented as the mean ± SD of eight animals. ^a–d^Values with different letters for a given week are significantly different from each other (Tukey’s-HSD multiple range *post hoc* test, *p* < 0.05). NC: Normal Control; DBC: Diabetic Control; DZL: Diabetic *Ziziphus mucronata* low dose (150 mg/kg bw); DZH: Diabetic *Ziziphus mucronata* high dose (300 mg/kg bw); DMF: Diabetic metformin; NZT: Normal *Ziziphus mucronata* toxicological dose (300 mg/kg bw).

**Figure 4.**
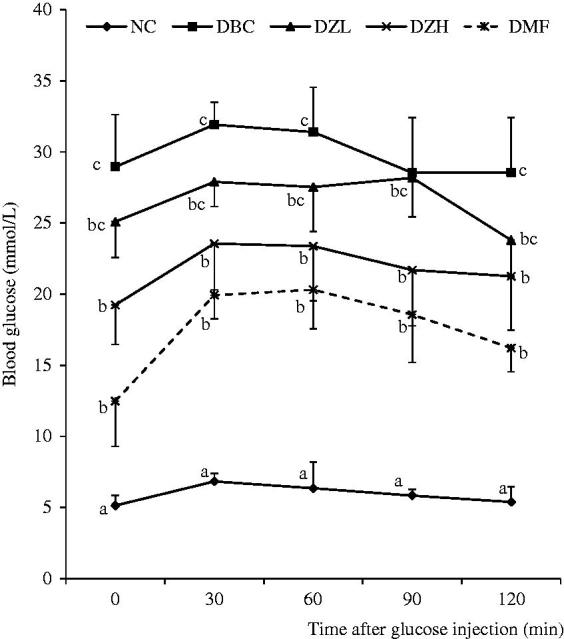
Oral glucose tolerance test (OGTT) for all groups of animals in the last week of experimental period. Data are presented as the mean ± SD of eight animals. ^a–c^Values with different letters for a given time are significantly different from each other (Tukey’s-HSD multiple range *post hoc* test, *p* < 0.05). NC: Normal Control; DBC: Diabetic Control; DZL: Diabetic *Ziziphus mucronata* low dose (150 mg/kg bw); DZH: Diabetic *Ziziphus mucronata* high dose (300 mg/kg bw); DMF: Diabetic metformin; NZT: Normal *Ziziphus mucronata* toxicological dose (300 mg/kg bw).

During the 4-week intervention period, feed and fluid intake as well as body weight gain was not significantly (*p* < 0.05) different between DBC, DZL and DZH groups (Figures 1 and 2). The ZMBF treatment also did not affect feed and fluid intake of normal rats. Also, there was no significant difference between the NFBG levels of DZL and DBC groups throughout the experiment except for a sharp decline in the NFBG level of DZL group after the first week of treatment, which was not subsequently sustained. On the other hand, the DZH group recorded a significantly lower (*p* < 0.05) NFBG level than the DBC group during the entire experimental period (Figure 3). The data for the OGTT are shown in Figure 4. Although the results were not significantly different (*p* > 0.05) but better glucose tolerance ability was observed in the DZL group compared to the DBC group. Furthermore, DZH group had a significantly (*p* < 0.05) better glucose tolerance ability compared to the DBC and DZL groups.

Serum insulin concentrations and β-cell functions (HOMA-β) were significantly lower (*p* < 0.05) while serum fructosamine concentrations and peripheral insulin resistance (HOMA-IR) were significantly higher in the DBC group compared to the NC group (Table 1). However, the ZMBF treated diabetic groups had significantly higher (*p* < 0.05) serum insulin concentrations but statistically similar β-cell functions, insulin resistance and serum fructosamine concentrations compared to the DBC group. The serum level of AST was neither affected by the induction of T2D nor the ZMBF treatments but serum ALT, ALP and urea levels were significantly elevated (*p* < 0.05) in the DBC group compared to the NC group. However, ZMBF treatments relatively increased the serum ALT level and reduced the serum ALP and urea levels in diabetic rats (Table 1). No significant differences were observed between the NC and NZT groups for all these parameters. Relatively lower serum creatinine concentration was observed in the DBC group, which was boosted in the DZL and DZH groups.

**Table 1.** Serum insulin and fructosamine concentrations, indices of hepatic and renal damage as well as computed HOMA-IR and HOMA-β scores for different animal groups at the end of the experimental period.

	NC	DBC	DZL	DZH	DMF	NZT
Insulin (pmol/L)	153.12 ± 9.91^bc^	64.20 ± 29.93^a^	148.24 ± 33.04^b^	131.37 ± 35.10^b^	119.19 ± 13.00^b^	165.73 ± 18.96^bc^
Fructosamine (μmol/L)	196.33 ± 5.57^a^	258.40 ± 10.92^ab^	258.60 ± 19.15^ab^	234.20 ± 20.02^ab^	253.83 ± 30.36^ab^	189.85 ± 61.02^a^
HOMA-IR	4.96 ± 1.05^a^	16.24 ± 5.65^b^	10.75 ± 5.73^b^	9.83 ± 4.51^b^	9.84 ± 3.78^b^	5.12 ± 0.70^a^
HOMA-β	304.07 ± 53.72^c^	9.68 ± 5.24^a^	24.90 ± 14.54^a^	13.62 ± 7.94^a^	54.85 ± 27.51^ab^	253.44 ± 30.98^c^
AST (U/L)	88.00 ± 14.88	73.60 ± 7.63	91.20 ± 26.30	71.80 ± 6.64	88.66 ± 17.87	89.16 ± 15.49
ALT (U/L)	56.25 ± 12.55^a^	77.75 ± 4.57^ab^	81.80 ± 30.02^ab^	88.25 ± 12.78^b^	63.33 ± 18.18^a^	57.33 ± 15.90^a^
ALP (U/L)	188.80 ± 19.86^a^	898.60 ± 174.49^bc^	578.00 ± 163.45^b^	510.50 ± 94.38^b^	472.00 ± 74.99^b^	214.42 ± 78.21^a^
Urea (mg/dL)	48.00 ± 6.16^a^	93.00 ± 16.40^ac^	67.80 ± 12.69^ab^	59.25 ± 22.93^a^	42.00 ± 11.78^a^	47.66 ± 9.00^a^
Creatinine (μg/dL)	582.00 ± 70.49^a^	485.00 ± 40.41^a^	563.33 ± 51.63^a^	550.00 ± 74.38^ab^	675.00 ± 95.23^ab^	471.42 ± 49.13^a^

Data are presented as the mean ± SD of eight animals.

a–c Values with different letters along a row are significantly different from each other (Tukey’s-HSD multiple range *post hoc* test, *p* < 0.05). NC: Normal Control; DBC: Diabetic Control; DZL: Diabetic *Ziziphus mucronata* low dose (150 mg/kg bw); DZH: Diabetic *Ziziphus mucronata* high dose (300 mg/kg bw); DMF: Diabetic metformin; NZT: Normal *Ziziphus mucronata* toxicological dose (300 mg/kg bw); HOMA-IR: Homeostatic Model Assessment – Insulin Resistance; HOMA-β: Homeostatic Model Assessment – Beta-cell function; AST; Aspartate transaminase; ALT: Alanine transaminase; ALP: Alkaline phosphatase.

The data for the liver weights and glycogen concentration of the experimental animals are presented in Table 2. Relative liver weight (liver weight relative to body weight) was significantly higher in the DBC and DZL groups compared to the NC group with no significant difference between the DBC and ZMBF treated diabetic groups. Conversely, liver glycogen was significantly depleted (*p* < 0.05) in the DBC group but the ZMBF treatment significantly (*p* < 0.05) boosted the liver glycogen reserves in the DZH group only.

**Table 2.** Liver weights and liver glycogen concentrations in different animal groups at the end of the experimental period.

	NC	DBC	DZL	DZH	DMF	NZT
Liver weight (g)	11.80 ± 2.35	10.98 ± 1.96	10.33 ± 1.96	10.28 ± 1.22	10.39 ± 1.73	11.94 ± 1.87
Relative liver weight (%)	3.28 ± 0.33^a^	3.96 ± 0.15^b^	4.01 ± 0.33^b^	3.81 ± 0.14^ab^	3.67 ± 0.12^a^	3.39 ± 0.43^a^
Liver glycogen (mg/g tissue)	3.53 ± 0.83^b^	1.54 ± 0.13^a^	2.20 ± 0.86^a^	2.40 ± 0.54^ab^	2.65 ± 0.38^ab^	2.97 ± 1.02^ab^

Data are presented as the mean ± SD of eight animals.

a–b Values with different letters along a row are significantly different from each other (Tukey’s-HSD multiple range *post hoc* test, *p* < 0.05). NC: Normal Control; DBC: Diabetic Control; DZL: Diabetic *Ziziphus mucronata* low dose (150 mg/kg bw); DZH: Diabetic *Ziziphus mucronata* high dose (300 mg/kg bw); DMF: Diabetic metformin; NZT: Normal *Ziziphus mucronata* toxicological dose (300 mg/kg bw).

Relative liver weight is the ratio of the liver weight of an animal to the total body weight of which was calculated using the following formula:.

Relative liver weight = (liver weight in gram/body weight in gram) × 100.

Table 3 presents the serum lipid concentrations at the end of the experimental period. Serum total cholesterol and LDL-cholesterol were not significantly different among the groups of rats. However, the DBC group recorded a significantly lower level of HDL-cholesterol, which was increased in the ZMBF-treated diabetic groups. Serum triglyceride concentration was significantly higher in the DBC group compared to the NC and NZT groups, but the DZL group had a relatively lower and the DZH group had a relatively higher serum triglycerides compared to the DBC group.

**Table 3.** Serum lipid profiles in different animal groups at the end of the experimental period.

	(mg/dL)					
Serum lipids	NC	DBC	DZL	DZH	DMF	NZT
Total cholesterol	66.25 ± 6.39	76.25 ± 10.62	61.00 ± 9.45	79.25 ± 17.25	74.63 ± 12.10	80.66 ± 11.28
HDL cholesterol	31.00 ± 9.55^b^	19.80 ± 1.64^a^	28.50 ± 5.24^ab^	26.00 ± 9.27^ab^	28.14 ± 5.39^ab^	32.42 ± 8.48^b^
LDL cholesterol	19.85 ± 5.35	27.45 ± 4.34	18.60 ± 8.47	16.50 ± 6.90	25.98 ± 7.43	30.57 ± 8.48
Triglycerides	102.50 ± 27.49^a^	184.75 ± 40.96^b^	113.50 ± 30.12^a^	198.40 ± 7.50^b^	120.13 ± 27.42^a^	92.00 ± 14.22^a^

Data are presented as the mean ± SD of eight animals.

a–b Values with different letters along a row are significantly different from each other (Tukey’s-HSD multiple range *post hoc* test, *p* < 0.05). NC: Normal Control; DBC: Diabetic Control; DZL: Diabetic *Ziziphus mucronata* low dose (150 mg/kg bw); DZH: Diabetic *Ziziphus mucronata* high dose (300 mg/kg bw); DMF: Diabetic metformin; NZT: Normal *Ziziphus mucronata* toxicological dose (300 mg/kg bw); HDL: high-density lipoproteins; LDL: low-density lipoproteins.

**Figure 5.**
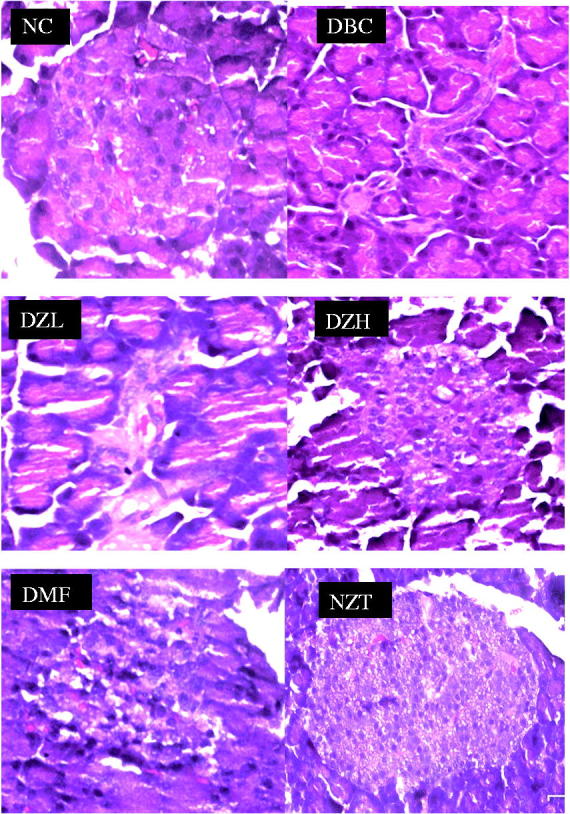
Representative sections of histopathological examinations of the pancreas from different animal groups. The NC group had higher number of cells per islet while the DBC and DZL group had smaller pancreatic islets with very few cells. The DZH had relatively higher number of cells per islet compared to DBC and DZL groups but far lower than the NC group. NC: Normal Control; DBC: Diabetic Control; DZL: Diabetic *Ziziphus mucronata* low dose (150 mg/kg bw); DZH: Diabetic *Ziziphus mucronata* high dose (300 mg/kg bw); DMF: Diabetic metformin; NZT: Normal *Ziziphus mucronata* toxicological dose (300 mg/kg bw).

Histopathological examination of the pancreatic sections revealed a reduction in the size of pancreatic islets as well as the number of β-cells per islet in the DBC group compared to the NC group. Insignificant difference was observed between DZL group and the DBC group. However, the DZH group had slightly higher number of β-cells per islet than the DBC group (Figure 5). The NZT group had similar pancreatic islets and the number of cells per islet with the NC group.

## Discussion

The role of medicinal plants in the health care delivery systems of many parts of the world has been duly recognized (Koehn & Carter 2005). However, the traditional use of a plant in the traditional management of a disease might not necessary be a reflection of its efficacy. This study reveals that the butanol fraction of *Z. mucronata* root, a plant traditionally used for the treatment of diabetes (Etuk et al. 2010) elicited anti-hyperglycemic effects but does not appreciably ameliorate the T2D-associated complications in a rat model.

The potent antioxidative activity of the *Z. mucronata* root ethanol extract (Ibrahim et al. 2012) prompted us to subject it to further studies. Solvent-solvent fractionation of the ethanol extract led us to a butanol fraction with potent α-glucosidase and α-amylase inhibitory activity (data not shown), which was subjected to an *in vivo* study in a T2D model of rats. Polyphagia and polydipsia with concomitant reduction of body weight are major symptoms of diabetes mellitus (American Diabetes Association 2007), which were also evidently observed in the diabetic groups of our experiment. These parameters are usually linked to prolong and stable diabetic condition (Islam 2011b). In our study, the treatment with ZMBF did not ameliorate the T2D-induced polyphagia, polydipsia and weight loss possibly because other diabetic complications, especially metabolic parameters, were not significantly affected by the fraction. On the other hand, metformin also could not completely ameliorate the T2D-induced polyphagia, polydipsia and weight loss but its effect on these parameters was better than ZMBF.

Elevated fasting or postprandial hyperglycemia is the hallmark of T2D and thus, maintaining glucose homeostasis provides a rationale for preventing the detrimental effects of hyperglycemia and its associated complications (Gin & Rigalleau 2000). At a dose of 300 mg/kg bw, the metformin and ZMBF displayed appreciable anti-hyperglycemic and insulinotropic effects, improved glucose tolerance without reversing the T2D-induced β-cell dysfunction and damage to the pancreatic architecture. Taken together, it appears that the fraction did not protect β-cell from destruction but potentiate the residual β-cells to secrete more insulin, which consequently might promote more glucose uptake by the cells. However, the inability of the fraction to decrease peripheral insulin resistance and at the same time, achieved significant anti-hyperglycemia suggests the involvement of another mechanism probably an extra pancreatic retardation of intestinal glucose production. This hypothesis is supported by the potent α-glucosidase and α-amylase inhibitory actions displayed by the ZMBF. On the other hand, the failure of the fraction to protect against pancreatic damage caused by the disease was contrary to our expectations. This is because the fraction had potent antioxidative activity and the protection against pancreatic damage is usually mediated through antioxidative dependent mechanisms because oxidative stress is an important contributor to pancreatic β-cell damage in T2D (Huang et al. 2011). We thus speculated that *in vivo* xenobiotic metabolism has transformed the bioactive antioxidative components to inactive forms.

Another important feature of experimentally induced diabetes is a reduction in the liver glycogen level (Islam 2011b), which is caused by the modulation of glycogen synthase and glycogen phosphorylase activities. Previous studies have demonstrated that a number of plant materials (Habibuddin et al. 2008; Jain et al. 2010) elicited an antidiabetic activity partly through stimulation of hepatic glycogenesis. Thus, the significantly higher liver glycogen content recorded in the DZH group compared to the DBC group indicated that the anti-hyperglycemic activity of the ZMBF at 300 mg/kg bw was mediated not only by stimulating insulin secretion and/or retarding carbohydrates digestion, but also possibly by increasing hepatic glycogen synthesis.

Lipids play an important role in the pathogenesis of experimentally induced diabetes and the level of serum lipids is usually elevated at diabetic state, which is a risk factor for T2D associated cardiovascular diseases (Farook et al. 2011). Although the results were not significant, the serum total cholesterol and LDL cholesterol tend to increase in the DBC group compared to the NC group. However, significant hypertriglyceridemia was observed in the DBC group compared to the NC group. Unfortunately, the ZMBF treatments did not appreciably ameliorate the disease-induced alterations in these lipids. In fact, the treatment at 300 mg/kg bw displayed hyperlipidemic tendencies in diabetic, but not in normal rats. Although a number of plant-derived phytochemicals have been shown to possess hypolipidemic activity in experimental diabetes (Islam & Choi 2007; Kumar et al. 2012; Boudjelal et al. 2012), findings from our study revealed that ZMBF contains some hyperlipidemic phytochemicals and therefore could be hazardous when treating cardiovascular diseases associated with T2D. Other indices of diabetic complications also indicated that the T2D caused a significant increase in serum levels of ALT, ALP and urea. However, despite the anti-hyperglycemic effects of the fraction, a close look at the results of these biomarkers of hepatic and renal functions also indicates that ZMBF treatments did not appreciably ameliorate the hepatic and renal damage caused by diabetes. Therefore, a further study is required in order to understand the basis of these observations. Apart from assessing renal damage, creatinine is also a predictor for T2D and insulin resistance, and previous studies have demonstrated lower serum creatinine level in T2D human subjects compared to normal individuals (Harita et al. 2009; Hjelmesæth et al. 2010). It is thus plausible to suggest that the relatively lower insulin resistance (HOMA-IR) of the ZMBF treated diabetic groups compared to DBC group is responsible for the relatively elevated serum creatinine levels recorded in these groups.

On a general note, we observed that ZMBF treatment at 150 mg/kg bw was not able to show antidiabetic activity for most of the parameters measured in this study. This might indicate that the amount of ZMBF has to reach a threshold level before the antidiabetic activity could be manifested.

We concluded that ZMBF, at a dose of 300 mg/kg bw, had anti-hyperglycemic activity which is mediated possibly through stimulating insulin secretion, retarding carbohydrates digestion and/or increasing hepatic glycogen synthesis. However, the fraction is not significantly effective in alleviating most of the T2D-associated complications. Our future work will focus on detailed studies on the molecular mechanism(s) for the observed results as well as the phytochemistry of the fraction.
